# Polycystic Ovary Syndrome Accompanied by Hyperandrogenemia or Metabolic Syndrome Triggers Glomerular Podocyte Injury

**DOI:** 10.3390/diagnostics14192197

**Published:** 2024-10-01

**Authors:** Kagan Gungor, Nur D. Gungor, Onder Celik, Aynur Ersahin, Nilufer Celik, Meltem Yardim, Arzu Yurci, Murat Kobaner, Ivan Ilkov Maslarski

**Affiliations:** 1 Department of Endocrinology, Istanbul Medeniyet University, Göztepe Prof. Dr. Süleyman Yalçın City Hospital, 34722 Istanbul, Turkey; 2 Department of Obstetrics and Gynecology, Bahcesehir University Goztepe Medicalpark Hospital, 34732 Istanbul, Turkey; nurkaganece@gmail.com (N.D.G.); aynur.ersahin@hotmail.com (A.E.); 3 Department of Obstetrics and Gynecology, Private Clinic, 64000 Usak, Turkey; 4 Department of Biochemistry, Behcet Uz Children’s Hospital, 35210 Izmir, Turkey; nilufercelik35@hotmail.com; 5 Department of Medical Biochemistry, Yerkoy State Hospital, 66900 Yozgat, Turkey; meltem_yardim@hotmail.com; 6 In Vitro Fertilization (IVF), Andrology and Genetics Center, Memorial Bahcelievler Hospital, 34180 Istanbul, Turkey; arzuyurci@yahoo.com; 7 Department of Urology, Yuregir State Hospital, 01415 Adana, Turkey; muratkobaner@gmail.com; 8 Department of Anatomy, Histology, Pathology, and Forensic Medicine, Faculty of Medicine, University of Sofia “St. Kliment Ohridski”, 1407 Sofia, Bulgaria; maslarsky@gmail.com

**Keywords:** PCOS, glomerular damage, podocyte, metabolic syndrome, hyperandrogenemia

## Abstract

**Objective:** To determine whether the urinary excretion of podocyte degradation products varies according to PCOS phenotype and metabolic syndrome (MetS). **Methods:** The concentrations of podocalyxin (PDX) and nephrin, chronic markers of podocyte damage, and neutrophil gelatinase-associated lipocalin (NGAL), a marker of acute glomerular damage, were analyzed in the morning urine samples of 50 PCOS patients and 50 healthy controls matched by age and BMI. Albuminuria was assessed by calculating the urine albumin–creatinine ratio (uACR). **Results:** The PDX, nephrin and NGAL concentrations of PCOS participants were significantly higher than those of the control group. While PDX, nephrin and NGAL levels of classic phenotypes were similar, they were higher than ovulatory and non-hyperandrogenic phenotypes. Significant increases in urinary levels of each podocyte protein were detected in PCOS patients with MetS compared to patients without MetS. A positive significant correlation between podocyte proteins and BMI, systolic blood pressure, testosterone, glucose, HOMA-IR and uACR. After adjusting for age and BMI, podocyte proteins were an independent risk factor for microalbuminuria. The incidence of microalbuminuria in PCOS increased 6-fold compared to controls. The frequency of microalbuminuria was higher in classical phenotypes than in ovulatory phenotype. The frequency of microalbuminuria in PCOS patients with MetS was 6.5 times higher than in PCOS patients without MetS. **Conclusions:** In PCOS accompanied by hyperandrogenemia or metabolic syndrome, leakage of acute and chronic podocyte breakdown products into the urine becomes more pronounced.

## 1. Introduction

Podocytes are glomerular epithelial cells with foot-shaped protrusions called pedicles and are located in the outermost part of the glomerular basement membrane. Podocytes, their pedicles and the glomerular basement membrane form tight junctions together, keeping the passage of plasma proteins into the Bowman’s space under control [1,2,3]. In addition to their physical barrier properties, podocytes contribute to the maintenance of renal filtration barrier integrity by producing podocalyxin and nephrin proteins. Nephrin is a podocyte-derived transmembrane protein and is more specific to the slit diaphragm in the glomeruli [4]. Podocalyxin (PDX) is the main component of the glycocalyx, which forms a chemical barrier on the podocytes. PDX is localized to the plasma membrane of podocytes [1]. The polyanionic sialoglycoprotein structure allows podocalyxins to block the passage of negatively charged proteins across the glomerular barrier. Podocyte damage leads to the breakdown of the glomerular barrier on both a physical and chemical level, resulting in the leakage of plasma proteins into the urine. Podocyte damage results in the excretion of podocyte degradation products and podocyte-specific marker proteins into the urine [2,3,4]. While an increase in PDX and nephrin in the urine is a result of long-term glomerular damage, neutrophil gelatinase-associated lipocalin (NGAL) is an indicator of early kidney injury. Mature neutrophils are the main cells where NGAL, a member of the lipocalin superfamily, is stored [5,6]. NGAL plays a role in glomerular damage through the enzymatic activation of matrix metalloproteinase-9 and the inflammatory modulation of innate immunity [7,8].

As in primary glomerular diseases, the underlying pathology in systemic diseases with renal involvement, such as PCOS, may lead to inflammation, adhesion and scar formation in the glomerular barrier, leading to podocyte damage, impaired urinary filtration and protein leakage [3,9]. The high incidence of obesity, chronic inflammation, hyperandrogenemia, hyperinsulinemia, hypertension and dyslipidemia in PCOS makes these patients susceptible to metabolic syndrome and renal injury [9,10]. In addition to the metabolic and hormonal imbalance in PCOS, elevated proinflammatory cytokines and chemokines can cause split diaphragm damage and alterations in the expression and urinary passage of podocyte-specific marker proteins [9,10]. The fact that the risk of coronary artery and carotid artery involvement with advancing age in hyperandrogenemic PCOS patients is 2.7 times higher than in healthy controls has brought to the agenda that the glomerulo-capillary network may also be affected in PCOS [11]. The increase in blood pressure and glomerular filtration rate (GFR) as well as moderate proteinuria in female rats exposed to dihydrotestosterone (DHT) supports this view [12]. Moreover, the significant increase in focal segmental glomerulosclerosis and interstitial fibrosis in animals exposed to androgens for a long time constitutes important evidence that metabolic changes, hyperandrogenemia and chronic inflammation can cause podocyte damage in PCOS [13].

The postmitotic phenotype of podocytes exposes them to the destructive effects of many physical and chemical stressors. The glomerular damage caused by diabetes mellitus and systemic vascular diseases suggest that PCOS, which is accompanied by widespread endocrine and metabolic pathologies, may cause renal damage through a similar mechanism [9,10,11,12,13]. Hyperandrogenemia, insulin resistance, and vascular endothelial damage induced by PCOS may impair glomerular hemostasis and cause podocyte injury. In addition to proteinuria, analysis of podocyte-specific degradation products is critical to understanding the possible relationship between PCOS and podocyte injury. Since microalbuminuria is an indicator of podocyte damage, the probability of detecting podocyte proteins in the urine before or simultaneously with microalbuminuria is quite high [14,15]. Most studies have analyzed changes in urine albumin, creatinine, protein or GFR to detect possible endothelial damage due to PCOS [12,16]. Examining podocyte proteins in the urine samples of PCOS patients is a non-invasive way to gain information about the potential adverse effects of hormonal or metabolic changes on the glomerular micromilieu. There is no clinical study analyzing glomerular tuft degradation products in the urine samples of PCOS patients. Therefore, measuring the urinary excretion of podocyte proteins, which are indicators of glomerular basement membrane damage, may be useful in the prediction of renal injury in PCOS [15]. Altered expressions of urinary podocyte-specific proteins may provide clues about the long-term adverse effects of PCOS on the glomerular barrier. For this purpose, we measured the levels of podocyte surface glycoprotein podocalyxin and podocyte pedicle slit protein nephrin in urine samples of PCOS patients. We also measured urinary NGAL levels to determine whether PCOS had an acute effect on the glomerular barrier. Since the possibility of glomerular injury is higher in patients with PCOS accompanied by hyperandrogenemia or metabolic syndrome [13], the participants were grouped and analyzed in two different ways. In the first comparison, the effects of hyperandrogenemia (HA), ovulatory dysfunction (OD) and polycystic ovarian morphology (PCOM) on podocyte damage were investigated by dividing PCOS patients into phenotype A (HA+OD+PCOM), phenotype B (HA+OD), phenotype C (HA+PCOM) and phenotype D (OD+PCOM) according to the National Institutes of Health (NIH) consensus panel [17]. In the second comparison, PCOS patients were grouped as those with or without metabolic syndrome, providing an opportunity to test the possible adverse effects of hyperglycemia, dyslipidemia, body mass index and hypertension on podocyte damage. In both comparisons, in addition to microalbuminuria, the podocyte degradation products PDX, nephrin and NGAL concentrations were measured in spot urine samples.

## 2. Materials and Methods

This cross-sectional study was approved by the Clinical Research Ethics Committee of the Bozok University Faculty of Medicine (Ethical approval number: 2017-KAEK-189_2023.02.17_1, date: 17 February 2023). Consent was obtained from each subject after a full explanation of the purpose and nature of all procedures used. Participants were selected from patients who applied to Göztepe Medicalpark Hospital’s IVF unit between February 2023 and September 2023. A total of 50 participants diagnosed with PCOS according to the European Society for Human Reproduction and Embryology/American Society for Reproductive Medicine (ESHRE/ASRM) diagnostic criteria were included in the study [18]. Fifty normotensive women, who were matched with the PCOS participants in terms of age and BMI, were taken as the control group. Both the PCOS and control groups consisted of 21–35-year-old participants with a BMI of 18.5–24.9 kg/m^2^. They were required to be free of cardiovascular disease, type 2 diabetes mellitus and other endocrine pathologies and have no history of renovascular disease.

PCOS was defined as having at least two of the criteria for hyperandrogenemia (HA), ovulatory dysfunction (OD) and polycystic ovarian morphology (PCOM). PCOS patients were subjected to two separate classifications to investigate the possible effects of phenotypic and metabolic variables on podocyte damage. In the first classification, participants were divided into four phenotypes as follows according to the 2012 NIH consensus panel [17]. Phenotype A: HA+OD+PCOM (n = 19); phenotype B: HA+OD (n = 15); phenotype C: HA+PCOM (n = 9) and phenotype D: OD+PCOM (n = 7). Dividing patients into phenotypes allowed us to test the effects of HA, OD, and PCOM on podocyte injury. In the second classification, patients were divided into two groups according to whether they had a metabolic syndrome or not. According to the National Cholesterol Education Program Adult Treatment Panel III, MetS is diagnosed if at least three or more of the five criteria are detected: a waist circumference > 35 inch, hypertension (>130 mm Hg systolic or >85 mm Hg diastolic or a high blood pressure requiring pharmacological treatment), a fasting triglyceride (TG) levels ≥ 150 mg/dL or requiring pharmacological treatment, a high-density lipoprotein (HDL) cholesterol level < 50 mg/dL and a fasting blood glucose ≥ 100 mg/dL [19]. The MetS classification provided us an opportunity to analyze the effects of hypertension, IR, obesity, serum lipid and glucose levels on podocyte damage. Patients with signs of a urinary tract or systemic infection, smokers, those with a history of nephritic or nephrotic syndrome, and diabetic patients were excluded from the study. Women with hyperandrogenism or ovulatory dysfunction due to endocrine etiologies other than PCOS were also excluded. Patients with a systemic disease with the possibility of renal involvement and those who used nephrotoxic, antihypertensive, insulin sensitizer, lipid-lowering or other hormonal drugs in the past six months were not included.

Anthropometric measurements such as body height, weight and waist circumference (WC) were performed on both groups. The height and weight of each participant standing without shoes were determined. Waist circumference was calculated as the distance between the lower rib margin and the iliac crest of the participants standing and breathing gently. BMI was calculated by dividing the subject’s weight (kg) by the square of the subject’s height (kg/m^2^). In phenotypes with ovulatory dysfunction, basal hormone, biochemical and urinary parameters were measured on days 2–5 of progesterone withdrawal bleeding. Samples were collected following at least 8–10 h of overnight fasting and stored at −20 °C until analysis. Blood samples were collected between days 2–5 of the spontaneous menstrual cycle in the control group and normo-ovulatory PCOS patients (phenotype C). Serum luteinizing hormone (LH), follicle stimulating hormone (FSH), estradiol, insulin and total testosterone levels were determined using the electro-chemiluminescence immunoassay (ECLIA) method with a Roche Cobas e602 (Roche Diagnostics GmbH, Mannheim, Germany) immunoassay analyzer. Fasting blood glucose (FBG), triglyceride (TG), high-density lipoprotein (HDL) and creatinine levels were measured on the AU 5800 (Beckman Coulter, Inc., Brea, CA, USA) autoanalyzer. FBG was measured by the hexokinase method, while creatinine levels were measured with the modified Jaffe method. The homeostasis model assessment of insulin resistance (HOMA-IR) was calculated with the following formula: insulin (μU/mL) × glucose (mg/dL)/405 [20].

### 2.1. Measurements of Urinary NGAL, Podocalyxin and Nephrin

Morning urine samples were used to evaluate podocyte damage. Vigorous exercise was prohibited 24 h before urine sampling [21]. Freshly obtained urine samples were collected in sterile containers. Subsequently, samples were centrifuged at 3500 rpm for 3 min, aliquoted and stored frozen until the day of analysis. Frozen urine samples were thawed on the day of the ELISA. Each participant’s urinary podocalyxin, nephrin and NGAL concentrations were measured using commercially available human kits using an ELISA. Kits required for the NGAL, nephrin and podocalyxin measurement were purchased from the Sunred Biotechnology Company, Shanghai, China. Each assay was performed in accordance with the manufacturer’s instructions specified in the kit catalog. The absorbances of the urine samples studied in accordance with the kit procedure were measured in a Bio-Tek ELx800 (BioTek Instruments, Winooski, VT, USA) device at a wavelength of 450 nanometers. The concentrations corresponding to all absorbances were calculated in ng/mL with the formula obtained with the help of the standard curve graph. The measuring range of the podocalyxin kit was 0.2–60 ng/mL and the minimum measurable level was 0.153 ng/mL. The standard curve range for the nephrin kit was 0.2–40 ng/mL. The minimum measurable nephrin level was 0.16 ng/mL. The measuring range of the NGAL kit was 12–3000 ng/mL, and the minimum measurable level was 10.51 ng/mL. The intra-assay CV value of each kit was <10%, while the inter-assay CV value was <12%.

### 2.2. Measurement of Spot Urine Albumin-to-Creatinine Ratio (uACR)

Albuminuria was assessed by calculating the albumin–creatinine ratio (uACR) in the morning, single-voided urine samples of all PCOS phenotype and control groups [22]. First-morning spot urine was preferred to avoid orthostatic proteinuria. A uACR cutoff ≥ 30 mg albumin/g Cr was used to define albuminuria. While normoalbuminuria was defined as uACR ≤ 30 mg albumin/g Cr excretion, microalbuminuria was defined as a uACR > 30 to 300 mg albumin/g Cr excretion.

### 2.3. Statistical Analysis

IBM SPSS Statistics Version 27.0 for Windows (IBM Corp., Armonk, NY, USA) was used for data analysis and GraphPad Prism 8.0 (GraphPad Software Inc., San Diego, CA, USA) for graph analysis. The data’s conformity to a normal distribution was assessed using the Shapiro–Wilk test. Data are shown as mean ± standard deviation and median (1st quartile-3rd quartile) for continuous variables according to the normality of the distribution. To compare two independent groups, a Student’s *t*-test was used if the data fit a normal distribution, and a Mann–Whitney *U* test was used if the data did not fit the normal distribution. A Kruskal–Wallis test was used to compare more than two independent groups. Pairwise comparisons were made with the Bonferroni correction method. Categorical variables are shown as numbers and percentages and comparisons between groups were analyzed by Fisher’s exact test. A Spearman correlation test was used for the relationship between variables. The correlation matrix heat map was drawn with R software 4.3.3 and the Metan R package [23]. Multivariate logistic regression analysis was performed to identify independent risk factors for microalbuminuria in PCOS patients after adjusting for age and BMI. Two-tailed *p* values of *p* < 0.05 were considered significant.

## 3. Results

**Figure 1:**
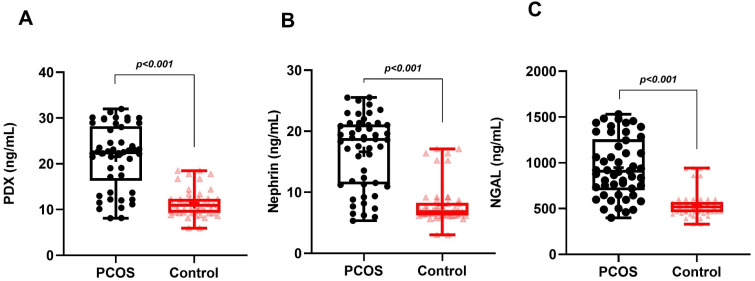
Graphical representation of urine levels of podocyte degradation products PDX (**A**), nephrin (**B**), and NGAL (**C**) in PCOS and control groups.

**Figure 2:**
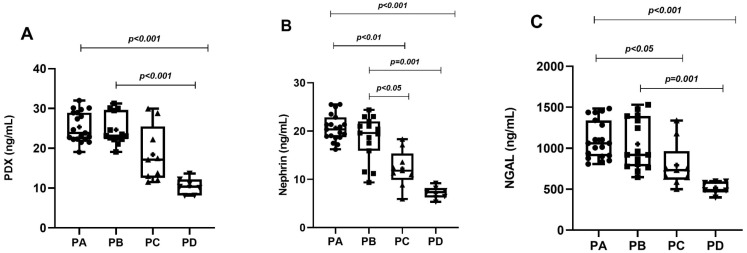
Graphical representation of urine levels of podocyte degradation products PDX (**A**), nephrin (**B**), and NGAL (**C**) according to phenotypes.

**Figure 3:**
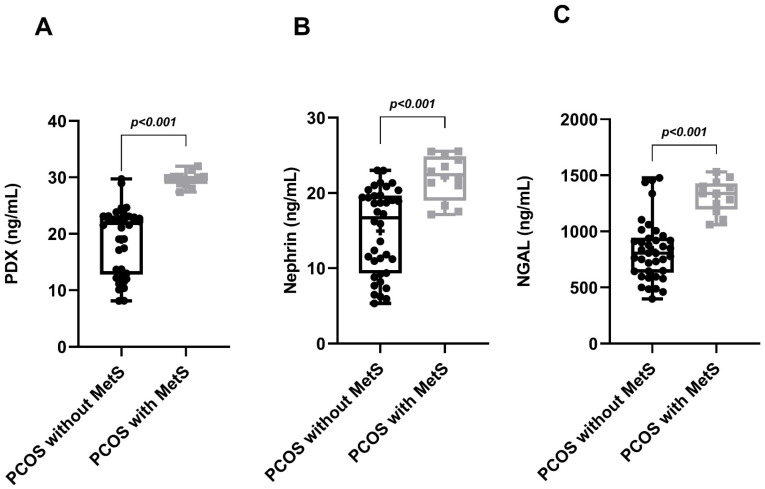
Graphical representation of urinary PDX (**A**), nephrin (**B**), and NGAL (**C**) levels when MetS is and is not a component of PCOS.

In the PCOS group, phenotype A was detected in 19 patients (38%), phenotype B in 15 patients (30%), phenotype C in 9 patients (18%) and phenotype D in 7 patients (14%). While MetS was detected in 12 patients in the PCOS group (24%), it was detected in only 3 patients (12%) in the control group. Age, BMI, WC, SBP, DBP, serum FSH, creatinine and TG levels of the PCOS and control groups were similar. Serum LH, total testosterone, HOMA-IR and FBG levels in the PCOS group were significantly higher than in the controls. The HDL-C levels of the PCOS group were significantly lower than the controls. The uACR, urinary PDX, nephrin and NGAL levels in PCOS participants were significantly higher than in the control group (Table 1, Figure 1). When we evaluated podocyte-specific proteins according to PCOS phenotypes, a statistically significant difference was observed between the phenotypes (Table 2, Figure 2). In the post-hoc Bonferroni correction analysis, it was observed that PDX values in phenotype A and phenotype B were significantly higher than in phenotype D (*p* < 0.05). After post-hoc Bonferroni correction, it was observed that the nephrin values in phenotype A and B were significantly higher than in the phenotype D and C groups (*p* < 0.05). After post-hoc Bonferroni correction, the NGAL value of phenotype A was also significantly higher than phenotypes D and C, while the NGAL value of phenotype B was recorded as higher than only phenotype D (*p* < 0.05). MetS had a frequency of 31.5% in phenotype A, 26.6% in B and 22.2% in C, while none of the participants in phenotype D had a MetS. There was a significant increase in urinary nephrin, PDX and NGAL levels in patients with PCOS accompanied by MetS compared to PCOS patients without MetS (Table 3, Figure 3).

**Figure 4:**
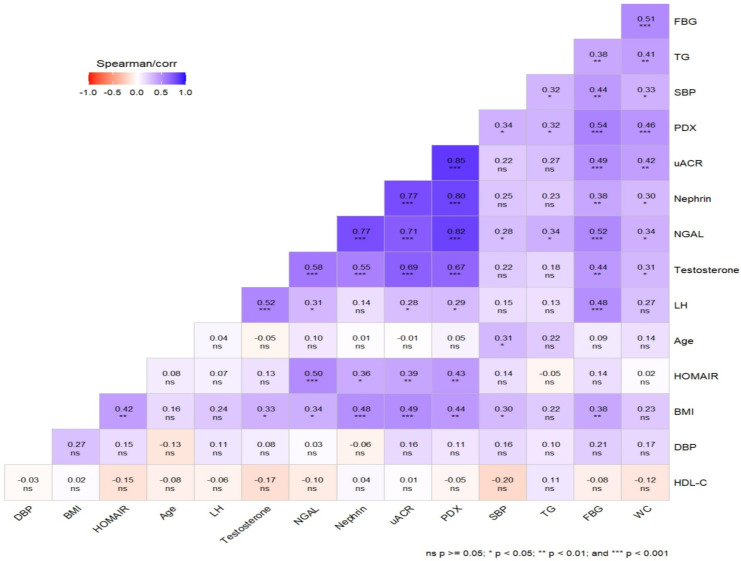
Graphical representation of the correlation matrices of variables. Blue colors indicate positive correlations, and red colors indicate negative correlations.

Positive, significant correlations were found between urinary PDX, nephrin, NGAL, BMI, WC, SBP, testosterone, glucose, HOMA-IR and uACR in the PCOS groups. Unlike urinary nephrin, urinary PDX and NGAL were also positively correlated with serum LH and TG levels (Figure 4). No correlation was found between serum creatinine levels and podocyte degradation markers. Multivariate logistic regression analysis revealed that high urine PDX (95% confidence interval 1.184–2.889), nephrin (95% confidence interval 1.029–2.001) and NGAL levels (95% confidence interval 1.001–1.009) were independent risk factors of incident microalbuminuria in PCOS patients after adjusting for potential confounders, including age and BMI (Table 4). Key findings of the study are summarized in Table 5.

Microalbuminuria was detected in 12 of the 50 patients in the PCOS group and in 2 of the 50 patients in the control group (Table 1). We observed a 6-fold increase in the incidence of microalbuminuria in PCOS patients compared to healthy controls (24% vs. 4%, *p* = 0.008). When evaluated according to phenotype, we found that the frequency of microalbuminuria was higher in phenotypes A (36.8%) and B (26.7%) than in phenotype C (11.1%) (*p* < 0.05). Microalbuminuria was not detected in the participants in phenotype D (Table 2). The frequency of microalbuminuria in PCOS patients with MetS was approximately 6.5 times higher than in the PCOS group without MetS (66.7% vs. 10.5%, *p* < 0.001, Table 3, Figure 3).

## 4. Discussion

Polycystic ovary syndrome increases the risk of cardiovascular diseases through endothelial damage in the affected population due to an imbalance in metabolic and hormonal parameters [24,25]. Until 2001, microalbuminuria was used as a diagnostic criterion for MetS as a marker of glomerular basement membrane damage [26]. The removal of microalbuminuria, which was previously a diagnostic criterion for MetS, has brought about the use of new urinary markers in detecting the adverse renovascular effects of systemic diseases such as PCOS [19]. Podocytes are differentiated epithelial cells of the glomerular basement membrane that have lost their regenerative properties [1,2]. In addition to primary glomerular diseases, many systemic diseases with kidney involvement [9] can cause podocyte damage. Podocalyxin and nephrin are the most used podocyte-specific marker proteins to detect chronic damage in podocytes [1,2]. Neutrophil-derived NGAL is a urinary marker [5] responsible for the acute and enzymatic destruction of the glomeruli. This study presents the first data showing a significant increase in the podocyte degradation products PDX, nephrin and NGAL in the urine of PCOS patients compared to non-PCOS controls. The incidence of microalbuminuria in the PCOS group was 24% and was six times higher than in the control group (4%). The positive, significant correlation between uACR, PDX, nephrin and NGAL suggests that podocyte damage is associated with the urinary excretion of podocyte functional proteins and albumin. However, although microalbuminuria is seen in only 24% of PCOS patients, the detection of podocyte degradation products in a larger cohort of PCOS patients suggests that the passage of podocyte proteins into the urine begins before albumin. Consistent with this, it has been reported that podocyte degradation products begin to be excreted in the urine before microalbuminuria occurs [14]. Therefore, we suggest that measuring the shedding of podocyte-specific proteins in the urine is more sensitive than the presence of microalbuminuria in detecting glomerular damage at an early stage. In patients with PCOS accompanied by MetS, the observed 6.5-fold increase in microalbuminuria (66.7% vs. 10.5%) compared to MetS-free PCOS patients strongly suggests that MetS increases the occurrence of glomerular damage. However, the detection of microalbuminuria with a frequency of 10.5% in PCOS patients without MetS suggests that MetS is not an absolute condition for microalbuminuria.

Since the phenotype may modulate the long-term risk incidence of PCOS, urinary podocyte markers were determined by classifying patients according to the NIH criteria [17]. When we evaluated PCOS patients according to their phenotypes, urinary podocyte proteins showed significant increases in phenotypes A and B compared to phenotypes C and D. In phenotype D, urinary excretion rates of podocyte biomarkers were similar to the control group. Since hyperandrogenemia (HA) is the common parameter of both phenotypes A and B, which have high urinary excretion rates of podocyte proteins, we can suggest that hyperandrogenemia is a stimulating factor for glomerular damage. The fact that podocyte marker protein levels in non-hyperandrogenemic phenotype D are similar to the control group is important evidence supporting the contribution of HA to podocyte damage. A positive, significant correlation between total testosterone and podocyte marker proteins is another finding that supports the podocyte-damaging effect of HA. Similarly, the positive correlation between serum LH and urinary PDX and NGAL suggests that LH directly or indirectly induces podocyte destruction by stimulating androgen synthesis. Hyperandrogenemia can lead to podocyte damage by activating multiple mechanisms and pathways in the glomerular basement membrane. High systemic androgen levels lead to increased sodium reabsorption through activation of the renin–angiotensin–aldosterone system (RAAS), leading to hypertension and increased intra-renal pressure [27,28]. High intra-renal mechanical stress causes podocyte damage and effacement of the foot ridges, inducing the formation of focal segmental glomerulosclerosis [9,27,28]. Although the glomerular filtration rate and the nitrate and nitrite excretion decreased in animals exposed to androgens, the increase in interstitial fibrosis in the glomerular basement membrane supports the adverse effects of androgens that cause podocyte damage and segmental glomerulosclerosis [13]. In addition, the finding that men are more sensitive to kidney damage than women, there is increased expression of androgen receptors in the glomeruli, there is increased glomerular RAAS activation in the presence of androgens, there is a higher frequency of kidney dysfunction in androgenic PCOS models, and high GFR rates are reported in many PCOS patients all support the long-term adverse effects of androgens in podocyte injury [13,29,30,31,32].

The incidence of MetS is two to six times higher in PCOS patients of reproductive age compared to non-PCOS women [33,34,35]. A higher prevalence of MetS has been reported in those with the hyperandrogenic PCOS phenotype [36]. The MetS frequency of patients in the PCOS group was 24%. MetS was most frequently associated with the hyperandrogenemic phenotypes (A, B) and less frequently with the ovulatory phenotype (C). MetS was not detected in the non-hyperandrogenic phenotype (D). The fact that the common phenotypic component of phenotypes A, B and C, where the prevalence of MetS is most common, is hyperandrogenemia, supports the view that high hyperandrogenemia increases the prevalence of MetS [36]. PCOS patients with accompanying MetS showed significantly increased urinary nephrin, PDX and NGAL levels compared to PCOS patients without MetS. The positive correlation of podocyte proteins with SBP, glucose, WC and TG is strong evidence for the podocyte-damaging effect of MetS. In MetS, each parameter alone, or their cumulative effects, can create a glomerular damaging environment. The more than two-fold prevalence of pregnancy-induced hypertension and preeclampsia in PCOS patients compared to the healthy population [37] is important evidence showing that the combination of PCOS and MetS has a damaging effect on the glomerulus. The fact that urinary podocyte protein loss is higher in women with preeclampsia than in healthy pregnant women and the close relationship between podocyturia and proteinuria [15,38] suggest that PCOS may cause podocyte damage through syndrome-specific metabolic pathologies. In patients with MetS accompanying PCOS, adipose tissue-derived adipokines may stimulate sodium reabsorption and RAAS, leading to increased intra-renal pressure and hypertension. In addition, adipokines may initiate the development of segmental glomerulosclerosis by stimulating inflammation and leptin-mediated TGF-β release [39,40,41]. In addition to obesity and inflammation, hyperinsulinemia may mediate glomerular hypertrophy by increasing the renal filtration rate and hypertension [39]. High triglyceride levels, on the other hand, may lead to podocyte damage by inducing oxidative stress, macrophage accumulation and fibrinogenic cytokine production [9,19,39].

In summary, preventing PCOS-induced glomerular injury is as important as preventing the long-term complications of PCOS, such as diabetes mellitus and endometrial cancer. Considering the adverse effects of renal diseases on a patient’s quality of life and duration, it is critical to control and minimize the glomerular injury effects of PCOS. In this context, lifestyle changes and medical treatments aimed at reducing insulin resistance and androgen elevation can be applied to PCOS patients with a hyperandrogenic phenotype or metabolic syndrome to prevent possible renal damage. In addition to regular physical exercise, dietary programs aimed at reducing adiposity can regulate BMI and arterial blood pressure and prevent endothelial dysfunction and glomerulosclerosis.

## 5. Conclusions

Although the relatively small number of participants with PCOS is a limitation, this study is the first to show that podocyte degradation products are increased in the urine of women with PCOS and that this is associated with albuminuria and glomerular injury. Urinary PDX, nephrin and NGAL are closely related to MetS, HA and uACR. Increased urinary levels of both acute and chronic podocyte degradation products led us to consider the early onset of glomerular stress in PCOS patients, especially in those with hyperandrogenic or MetS comorbidities. Although most PCOS patients do not have obvious clinical and laboratory findings of renovascular pathology, it may be important to investigate urinary podocyte proteins to prevent the development of glomerular injury in the future. In addition to conventional analyses, such as microalbuminuria, serum creatinine and blood urea nitrogen, podocyte protein analyses may be an important precaution to support the early diagnosis of glomerular damage. Urine analysis of podocyte proteins may also provide more objective results because it minimizes the confounding effects of orthostatic proteinuria and exercise on microalbuminuria. Studies on the use of insulin sensitizers or antiandrogens in PCOS patients with podocyturia and proteinuria may help develop new approaches to prevent the negative effects of PCOS-induced endocrine and metabolic pathologies on glomeruli.

## Figures and Tables

**Table 1:** Demographic and laboratory characteristics of participants in the PCOS and control groups.

	PCOS (n = 50)	Control (n = 50)	*p* Values
Age (year)	23.5 (22–25)	24 (22–26)	0.989
BMI (kg/m^2^)	23.2 (22.1–24.0)	22,5 (21.15–24.75)	0.117
Waist circumference (cm)	85.22 ± 8.65	82.56 ± 6.49	0.085
SBP (mm/Hg)	112.70 ± 12.87	107.60 ± 10.89	0.035
DBP (mm/Hg)	75.00 (70.00–80.00)	70.00 (67.00–80.00)	0.132
FSH (U/L)	5.5 (5.0–6.0)	5.8 (5.10–6.25)	0.139
LH (U/L)	8.94 ± 1.60	6.54 ± 0.98	<0.001
T. testosterone (ng/dL)	37.66 ± 7.71	28.72 ± 5.05	<0.001
HOMA-IR	2.47 ± 0.48	1.59 ± 0.31	<0.001
HDL-C (mg/dL)	40.96 ± 6.42	46.28 ± 6.58	<0.001
Triglyceride (mg/dL)	121.50 (102.00–154.25)	114.00 (102.75–130.00)	0.072
FBG (mg/dL)	90.96 ± 16.07	83.16 ± 11.36	0.006
Creatinine (mg/dL)	0.82 ± 0.76	0.68 ± 0.19	0.183
Microalbuminuria, n (%)	12 (24%)	2 (4%)	0.008
Nephrin (ng/mL)	16.63 ± 5.93	7.95 ± 3.33	<0.001
PDX (ng/mL)	21.56 ± 6.86	11.37 ± 2.94	<0.001
NGAL (ng/mL)	906.51 (701.24–1255.12)	518.16 (460.30–572.04)	<0.001
uACR (mg/gCr)	6.00 (4.00–26.5)	3.00 (3.00–9.25)	<0.001

Data are given as mean ± standard deviation or median (1st quartile–3rd quartile) for continuous variables according to the normality of the distribution and as frequency (percentage) for categorical variables.

**Table 2:** Distribution of MetS, podocyte damage markers and albuminuria frequencies according to phenotypes.

	Phenotype A	Phenotype B	Phenotype C	Phenotype D
Variables	HA+OD+PCOM	HA+OD	HA+PCOM	OD+PCOM
N, (%)	19 (38%)	15 (30%)	9 (18%)	7 (14%)
MetS	6 (31.5%)	4 (26.6%)	2 (22.2%)	-
PDX (ng/mL)	23.86 (22.27–28.96) *	23.08 (22.19–29.49) *	13.72 (12.50–23.11)	10.38 (8.14–12.17)
Nephrin (ng/mL)	20.35 (18.71–22.86) *^,‡^	19.62 (15.90–22.02) *^,‡^	11.81 (9.89–15.35)	7.34 (6.24–8.18)
N-GAL (ng/mL)	1061.32 (901.51–1340.98) *^,‡^	918.63 (777.32–1391.88) *	732.600 (615.00–965.39)	488.95 (461.35–595.69)
Normoalbuminuria (uACR ≤ 30 mg/gCr)	12 (63.2%)	11 (73.3%)	8 (88.8%)	7 (100%)
Microalbuminuria (uACR > 30–300 mg/g Cr)	7 (36.8%) ^α^	4 (26.7%) ^α^	1 (11.1%)	-

Bonferroni adjusted *p* values were used. * *p* < 0.05 vs. phenotype D, ^‡^ *p* < 0.05 vs. phenotype C, ^α^ *p* < 0.05 vs. phenotype C.

**Table 3:** The effect of whether PCOS is accompanied by MetS on the frequency of podocyte markers and albuminuria.

	PCOS with MetS (n = 12)	PCOS without MetS (n = 38)	*p*
Nephrin (ng/mL)	22.44 (18.99–24.86)	16.72 (9.32–19.63)	<0.001
Podocalyxin (ng/mL)	29.97 (28.82–30.11)	21.86 (12.79–22.95)	<0.001
NGAL (ng/mL)	1339.55 (1195.10–1428.10)	806.03 (632.13–943.29)	<0.001
Microalbuminuria (uACR > 30–300 mg/gCr)	8 (66.7%)	4 (10.5%)	<0.001

Data are given as median (1st quartile–3rd quartile) for continuous variables and as frequency (percentage) for categorical variables.

**Table 4:** Logistic regression analysis of the effect of urine PDX, NGAL and nephrin levels on the incident of microalbuminuria after adjusting for age and BMI.

	Unadjusted		Adjusted ^(1)^	
	OR (95% CI)	*p*	OR (95% CI)	*p*
PDX (ng/mL)	1.653 (1.243–2.199)	<0.001	1.849 (1.184–2.889)	<0.01
Nephrin (ng/mL)	1.609 (1.153–2.244)	<0.01	1.435 (1.029–2.001)	<0.05
NGAL (ng/mL)	1.005 (1.002–1.007)	<0.01	1.004 (1.001–1.009)	<0.05

OR: odds ratio, CI: confidence interval, ^(1)^ adjusted for age and BMI.

**Table 5:** Summary of key findings.

PCOS increases urinary levels of acute (NGAL) and chronic podocyte damage markers (PDX and nephrin). The increase in podocyte breakdown products is more pronounced in the presence of hyperandrogenemia or metabolic syndrome. Podocyturia is accompanied by microalbuminuria, but no increase in creatinine. Podocyturia is more pronounced in classical phenotypes than in ovulatory and normoandrogenic phenotypes. Urinary podocyte breakdown products are an independent risk factor for microalbuminuria, independent of age and BMI. BMI, systolic blood pressure, testosterone, glucose, HOMA-IR and uACR stimulate podocyte breakdown.

## Data Availability

All data used and/or analyzed during the current study are presented within the study. There is no additional data to request.
